# The complete mitochondrial genome of *Panopea abrupta* (Myoida: Hiatellidae)

**DOI:** 10.1080/23802359.2016.1258338

**Published:** 2016-11-22

**Authors:** Mingjia Yu, Shengping Zhong, Shanjun Yang, Jianming Chen, Tusar T. Saha

**Affiliations:** a Engineering Research Center of Marine Biological Resource Comprehensive Utilization, Third Institute of Oceanography, State Oceanic Administration, Xiamen, Fujian, China;; b Department of Entomology, University of California, Riverside, CA, USA

**Keywords:** Mitochondrial genome, *Panopea abrupta*, Bivalves

## Abstract

The goeduck clam *Panopea abrupta* (Myoida: Hiatellidae) is one of the most important freshwater aquaculture species in China. In-spite of its economic importance, however, the genomic information of this species remains unavailable. In this study, we report the complete mitochondrial genome sequence of *P. abrupta* along with annotated and fully characterized mitochondrial genes. The genome was found to be 15,381 bp in length with a total of 38 genes (13 protein-coding, 22 transfer RNAs, and 2 ribosomal RNAs). The presence of a gene coding for ATPase subunit 8 was also noted. However, as expected in bivalves, the gene arrangements showed variations with that of the related species. This study adds to the repository of available mitogenomes of various Heterodonta and will greatly aid in future phylogenetic studies and species identification.

The genus *Panopea* includes large marine bivalves known as geoducks, is one of the largest amongst filter-feeding burrowing bivalves. These clams are renowned for their extreme longevity and are reported to reach a record age of 168 years (Bureau et al. 2002). The geoduck clam, being widely cultivated in Southeast Asia and southeast coastal region in China, has great market demand in China. The species is characterized by the presence of sawtooth in front of the shell, which resembles a hypertrophic meat pipe. In spite of its commercial importance, adequate information about the genetic structure of geoduck clam population is still missing. Here, we report the complete mitochondrial genome sequence of *P. abrupta*, wishing to assist in molecular identification of this species and help improve the phylogenetic classification of the family *Hiatellidae*.

The adult specimen of *P. abrupta* (TA00523) was collected from Guangdong province, China, and stored at −80 °C in Third Institute of Oceanography, State Oceanic Administration, Xiamen, China. The total genomic DNA was extracted from the muscle of the specimens using an SQ Tissue DNA Kit (OMEGA, Guangzhou, China) following the manufacturer’s protocol. DNA libraries (500bp insert) were constructed with the TruSeq NanoTM kit (Illumina, San Diego) and were sequenced (100 bp paired end reads) using HiSeq platform at Gene Denovo Company, China. CLC Genomics Workbench 7.0.3 (CLC bio, Boston, MA) was used for *de novo* assembly. Gene annotation was carried using MITOS (Bernt et al. 2013) and BLAST searches. Boundaries of structural and rRNA genes were resolved using other available bivalve mitochondrial sequences. The tRNA genes were identified by DOGMA (Wyman et al. 2004), MITOS, and t-RNA scan-SE Search Server (Lowe & Eddy 1997).

The complete mitogenome of *P. abrupta* was found to be 15,381 bp in length (GenBank accession no. KX494111), consisting of the usual set of 13 protein-coding, 22 tRNA, and 2 rRNA genes. The overall base composition of the mitogenome is: A 25.6%, T 38.78%, C11.34%, and G 24.27% which is similar, but slightly different from *P. generosa* and *P. globosa* (Bisbal-Pardo et al. 2014a,b). In total, 3688 amino acids were encoded by the 13 identified protein-coding genes in *P. abrupta* mitochondrial genome. Leucine (14.72%) was found to be the most frequent amino acid, while Glutamine (1.18%) was the least prevalent. All protein-coding genes were found to use the initiation codon ATG except for *COXI*, *CYTB*, and *COXIII* genes, where GTG, GTG, and ATA served as the initiation codon, respectively. Twenty-nine intergenic spacers were found, ranging from 1 to 301 bp in length with the highest length recorded between *COXI* and *COXII* genes. The total length of non-coding region was calculated to be 880bp. Intergenic region was lacking between the gene pairs- tRNA-His/tRNA-Glu, tRNA-Leu2/NAD1, 16s rRNA/ATP6, and 12s rRNA/COXIII in addition to the overlapping bases between the structural gene tRNA-Glu/tRNA-Ser2, NAD3/tRNA-Ille, and NAD5/NAD6 (Table 1). The detailed description of 38 mitochondrial gene sequences characterized in *P. abrupta*, has been shown in Table 1, and the phylogenetic tree was constructed using maximum-likelihood method (Figure 1). The complete mitochondrial genome sequence of *P. abrupta* adds to the number of sequenced mitogenomes within the subclass Heterodonta and will aid the detailed phylogenetic studies undertaken henceforth.

**Figure 1.**
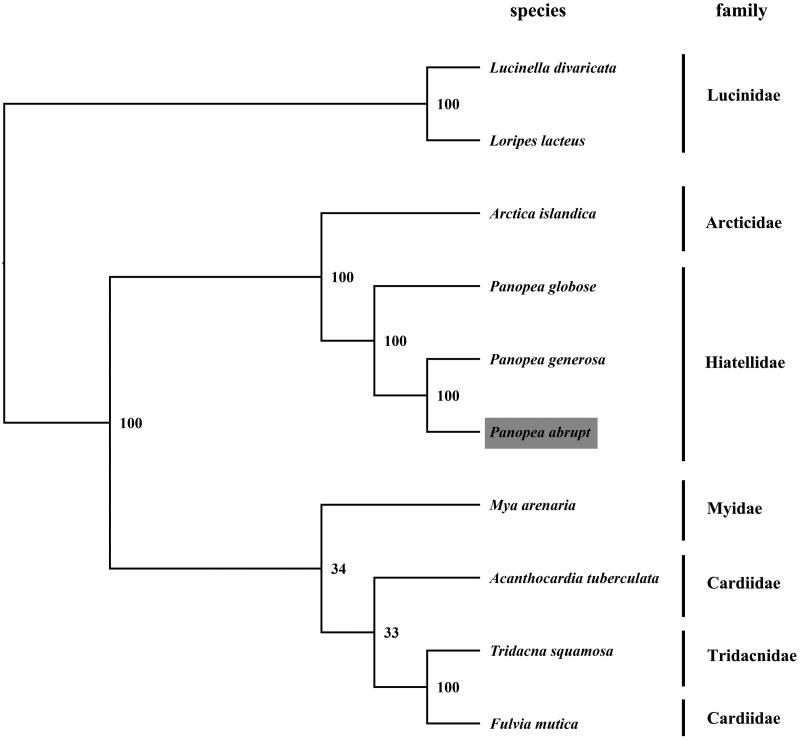
Phylogenetic tree of the complete mitogenomes. The phylogenic tree is constructed by maximum-likelihood method with 500 bootstrap replicates. The GenBank accession numbers are as follows: *P. globose* (NC_025636), *P. generosa* (NC_025635), *Mya arenaria* (NC_024738), *Lucinella divaricata* (NC_013275), *Loripes lacteus* (NC_013271), *Arctica islandica* (NC_022709), *Fulvia mutica* (NC_022194), *Tridacna squamosa* (NC_026558), and *Acanthocardia tuberculata* (NC_008452).

**Table 1.** Detailed description of the 38 mitochondrial genes in *P. abrupta*.

Gene	Position	Length(bp)	Start codon	Stop codon
*COXI*	1–1563	1563	GTG	TAG
*COXII*	1865–2602	738	ATG	TAA
*tRNA-Val*	2613–2677	65		
*tRNA-Thr*	2680–2745	66		
*tRNA-Tyr*	2815–2876	62		
*NAD4L*	2887–3177	291	ATG	TAG
*ATP8*	3230–3343	114	ATG	TAA
*NAD4*	3507–4694	1188	ATG	TAG
*tRNA-His*	4713–4758	46		
*tRNA-Glu*	4759–4822	64		
*tRNA-Ser2*	4818–4880	63		
*NAD3*	4884–5249	366	ATG	TAG
*tRNA-Ile*	5249–5315	67		
*tRNA-Asp*	5325–5385	61		
*tRNA-Lys*	5393–5452	60		
*tRNA-Leu2*	5455–5519	65		
*NAD1*	5520–6443	924	ATG	TAG
*tRNA-Leu*	6445–6509	65		
*tRNA-Asn*	6542–6609	68		
*NAD5*	6611–8335	1725	ATG	TAA
*NAD6*	8335–8859	525	ATG	TAG
*tRNA-Arg*	8865–8925	61		
*CYTB*	8928–10085	1158	GTG	TAA
*tRNA-Trp*	10095–10161	67		
*16S rRNA*	10250–11306	1057		
*ATP6*	11307–12014	708	ATG	TAA
*tRNA-Met*	12022–12085	64		
*12S rRNA*	12087–12946	860		
*COXIII*	12947–13735	789	ATA	TAG
*tRNA-Ser*	13741–13805	65		
*NAD2*	13807–14853	1014	ATG	TAA
*tRNA-Gln*	14867–14932	66		
*tRNA-Phe*	14941–15003	63		
*tRNA-Cys*	15018–15082	65		
*tRNA-Pro*	15105–15171	67		
*tRNA-Gly*	15182–15247	66		
*tRNA-Ala*	15263–15327	65		
